# Comparative assessment of the antirestenotic efficacy of two paclitaxel drug-eluting balloons with different coatings in the treatment of in-stent restenosis

**DOI:** 10.1007/s00392-015-0934-0

**Published:** 2015-11-05

**Authors:** Freek Nijhoff, Pieter R. Stella, Maartje S. Troost, Anouar Belkacemi, Hendrik M. Nathoe, Michiel Voskuil, Mariam Samim, Pieter A. Doevendans, Pierfrancesco Agostoni

**Affiliations:** Department of Cardiology, University Medical Center Utrecht, Utrecht, The Netherlands; Department of Cardiology, Isala Clinics, Zwolle, The Netherlands; Department of Cardiology, St. Antonius Hospital, Koekoekslaan 1, 3435 CM Nieuwegein, The Netherlands

**Keywords:** Drug-eluting balloon, In-stent restenosis, Paclitaxel, Percutaneous coronary intervention

## Abstract

**Background/objectives:**

Preclinical investigations have suggested that coating technology is crucial for the efficacy of drug-eluting balloons (DEB). Aim of this study is to compare the antirestenotic efficacy of two paclitaxel DEB with different coatings in the treatment of in-stent restenosis (ISR) by means of a morphological and functional assessment.

**Methods:**

In a single center, prospective, non-randomized study, the shellac-paclitaxel coated DIOR, and the urea-paclitaxel coated IN.PACT Falcon were compared in the setting of ISR. Quantitative angiography, fractional flow reserve (FFR), and optical coherence tomography (OCT) were performed at baseline, postprocedure and 6-month follow-up. Main endpoints were QCA, FFR and OCT-based parameters of restenosis.

**Results:**

Forty-five patients were included, 20 (44 %) received treatment with the DIOR and 25 (56 %) with the IN.PACT Falcon. Angiographic and device success were 100 and 90 % for the DIOR, and 100 and 92 % for the IN.PACT Falcon, respectively. After 6-months, in-segment late lumen loss (−0.03 ± 0.43 vs. 0.36 ± 0.48 mm, *p* = 0.014) and diameter stenosis (30.7 ± 16.2 vs. 41.3 ± 22.6 %, *p* = 0.083) were lower for the IN.PACT Falcon. FFR distal of the stent was significantly higher in the IN.PACT Falcon group (0.92 ± 0.07 vs. 0.84 ± 0.13, *p* = 0.029) and in-stent FFR gradient was lower (0.05 ± 0.05 vs. 0.13 ± 0.12, *p* = 0.002). Between postprocedure and follow-up, a 16 % decrease in neointimal volume was observed for the IN.PACT Falcon, while a 30 % increase was observed for the DIOR (*p* = 0.006).

**Conclusions:**

The IN.PACT Falcon DEB showed higher antirestenotic efficacy than the DIOR in the treatment of ISR, demonstrating that DEB with an excipient-based coating is not equally effective.

**Electronic supplementary material:**

The online version of this article (doi:10.1007/s00392-015-0934-0) contains supplementary material, which is available to authorized users.

## Introduction

Developments in stent design reduced the need for repeat revascularization after percutaneous coronary intervention (PCI). The lower revascularization rates following PCI are predominantly due to the introduction of drug-eluting stents (DES) which decreased the rate of in-stent restenosis (ISR) compared to bare-metal stents (BMS). Across different indications and lesion types, the incidence of ISR at one year has fallen below 5 % in new generation DES [1, 2]. Nevertheless, ISR still remains a significant problem due to the large numbers of patients that undergo PCI with stent implantation.

The challenging nature of ISR treatment is illustrated by the numerous strategies that have been evaluated over the years. Implantation of a DES appeared most effective, yielding better results than conventional balloon angioplasty alone [3, 4], cutting or scoring balloon treatment [5], bare-metal stenting [6] and brachytherapy [7]. More recently, the drug-eluting balloon (DEB) has been introduced as an alternative approach to ISR [8]. Randomized controlled trials (RCTs) have shown both clinical and angiographic non-inferiority of paclitaxel DEB compared to DES in BMS-ISR and DES-ISR [3, 9, 10]. Important advantage of DEB angioplasty in ISR is the avoidance of multiple layers of metal, providing more flexibility for future repeat interventions on the target lesion.

However, not all DEB may be equally effective in ISR [2]. The strength of DEB angioplasty resides in the suppression of neointimal hyperplasia, the main cause of recurrent ISR [11], by local delivery of an antiproliferative drug (paclitaxel) [12]. The magnitude of neointima inhibition depends on the ability of the DEB to create and sustain sufficient tissue concentrations of drug at the target lesion site. DEB coatings, designed to enhance the dissolution of paclitaxel from the balloon surface, are crucial to this process [13]. Differences in coating technology, i.e., the type of solvent, excipient and coating method, may lead to heterogeneity in the pharmacokinetic profile and thus antirestenotic efficacy among DEB [13–15]. This was previously observed in preclinical investigations and may be reflected in the ambiguous results among clinical studies investigating different DEB for identical indications [16, 17]. Data on head-to-head comparisons of DEB in the treatment of patients with ISR are scarce, however [18].

Aim of this study is to compare the antirestenotic efficacy of two established DEB with different coatings in the treatment of ISR. Comparative assessment has been performed by evaluation of DEB performance through serial morphological and functional assessment using quantitative coronary angiography (QCA), optical coherence tomography (OCT), and fractional flow reserve (FFR).

## Methods

### Study design and patient selection

This study is a post hoc analysis of a prospective, single center, and non-randomized study, which was originally designed to elucidate the mechanism of action of DEB in the setting of ISR. Patients with angina pectoris (both stable and unstable) or silent ischemia, who were scheduled for PCI because of ISR in a BMS or DES were regarded eligible. Exclusion criteria were: acute myocardial infarction, left main disease, ostial ISR (unfit for OCT evaluation), ISR located in a coronary bypass graft, recurrent ISR, presence of renal failure (creatinine ≥200 µmol/L), left ventricular ejection fraction ≤30 % and estimated life expectancy <12-months. The study was approved by the ethics committee of the University Medical Center Utrecht and conducted in compliance with the Declaration of Helsinki. All included patients provided signed informed consent.

### Interventional procedure

All study patients were treated with daily acetylsalicylic acid (80–100 mg) and clopidogrel (75 mg). If not pre-treated, a loading dose of clopidogrel (300–600 mg) was administered before the procedure. Procedural anticoagulation (aimed activated clotting time ≥250 s) was established by intravenous heparin. Administration of glycoprotein IIb/IIIa inhibitors was left at the physicians discretion. After the procedure, acetylsalicylic acid was continued indefinitely and clopidogrel was continued for 1 months.

Treatment of the target lesion was performed by sequential standard balloon predilatation and DEB dilatation. Standard balloons were sized with a 0.9:1 balloon-to-index-stent-diameter ratio, with a length shorter than the intended DEB, and inflated at high pressures (12–19 atm). DEBs were sized with a 1.1:1 balloon-to-index-stent-diameter ratio and inflated at lower pressures (6–10 atm) for 60 s. DEB length was selected to avoid geographic miss (i.e., the DEB should extend ≥5 mm proximal and distal of the predilatation balloon) and undesired DEB overlap in case of multiple DEB use in long lesions. Device characteristics are summarized in Table 1 and elaborated on in the supplementary methods (Online Resource 1). Postdilatation with standard balloons was left at the physicians discretion. Additional stenting was performed in case of stent edge dissection or residual stenosis. Before commencing PCI and directly afterwards, FFR and OCT were performed consecutively. Predilatation with a small diameter (1.5 or 2.0 mm) standard balloon was allowed in case the target lesion could not be crossed with the FFR guidewire or the OCT catheter at baseline. A detailed methodological description of the acquisition and offline analysis of QCA, OCT and FFR data is provided in the supplementary methods (Online Resource 1).

**Table 1:** Device characteristics of the drug-eluting balloons

	DIOR	IN.Pact Falcon
Manufacturer	EuroCor GmbH, Germany	Medtronic Vascular Inc., USA
Balloon type	Semi-compliant	Semi-compliant
Balloon diameters available, mm	2.0, 2.25, 2.5, 2.75, 3.0, 3.5, 4.0	2.0, 2.25, 2.5, 2.75, 3.0, 3.25, 3.5, 3.75, 4.0
Balloon lengths available, mm	15, 20, 25, 30	14, 20, 30, 40
Coated drug	Paclitaxel	Paclitaxel
Drug	Paclitaxel	Paclitaxel
Loading dose	3 µg/mm^2^	3 µg/mm^2^
Excipient	Shellac	Urea
Coating procedure	Micro-pipetting	Not specified
Inflation time, s	30–60	30–60
CE marking	2007	2009

*CE* Conformité Européene

### Follow-up and endpoints

Angiographic follow-up was scheduled per protocol at 6 months, unless indicated earlier on clinical grounds. Clinical follow-up was obtained simultaneously with angiography or by telephone interviews at 6 months. All clinical events were documented after careful examination of relevant hospital files. Antirestenotic efficacy, the outcome of interest, refers to the potency of the DEB to inhibit neointimal growth, not to the prevention of clinical restenosis per se. Main endpoints of this study were angiographic in-segment late luminal loss (LLL) and diameter stenosis, percentage changes in FFR and OCT parameters, and clinical outcomes according to the Academic Research Consortium criteria. Please see the Supplementary Methods (Online Resource 1) for endpoint definitions.

### Statistical analysis

All data were analyzed using IBM SPSS Statistics software version 20 (IBM Corp., Armonk, NY). Continuous variables were presented as mean ± standard deviation or medians (interquartile range), as appropriate. Categorical variables were reported as counts and percentages. Comparison of continuous variables was performed using the Mann–Whitney *U* test, considering small group sizes. Categorical variables were compared by means of Chi Square or Fisher’s Exact Test. For the most important outcomes, linear regression was used to adjust for differences in relevant baseline and procedural variables between both groups. A two tailed *p* value ≤ 0.05 was regarded statistically significant.

## Results

Forty-five patients were included in this study between August 2009 and April 2013. Twenty-five patients (56 %) were treated with the IN.PACT Falcon (results of this cohort have been previously published) [19] and subsequently 20 (44 %) with the DIOR DEB. Baseline characteristics of patients and lesions showed a high level of resemblance between both treatment groups (Table 2). A higher incidence of hyperlipidemia was observed in the DIOR group (*p* = 0.01). The patterns of ISR were comparable between the groups. The majority comprised BMS-ISR, while DES-ISR was present in 5 patients (25 %) in the DIOR and three patients (12 %) in the IN.PACT Falcon group (*p* = 0.44).

**Table 2:** Baseline patient and lesion characteristics

	DIOR (*n* = 20)	IN.Pact Falcon (*n* = 25)	*p* value
Patient characteristics			
Age, years	66.6 ± 10.27	65.3 ± 9.69	0.66
Male gender	14 (70)	17 (68)	0.89
Diabetes mellitus	4 (20)	6 (24)	0.75
Hypertension	11 (55)	14 (56)	0.95
Hyperlipidemia	13 (65)	7 (28)	0.01
Current smoker	3 (15)	4 (16)	0.93
Family history of cardiovascular disease	10 (50)	12 (48)	0.89
Previous myocardial infarction	10 (50)	14 (56)	0.69
Previous coronary artery bypass grafting	3 (15)	3 (12)	0.77
Lesion characteristics			
Target vessel			0.22
Left anterior descending	10 (50)	12 (48)	
Ramus circumflex	6 (30)	3 (12)	
Right coronary artery	4 (20)	10 (40)	
Pattern of restenosis^a^			0.47
Focal body	8 (40)	5 (20)	
Multifocal	0	0	
Diffuse in-stent	8 (40)	15 (60)	
Proliferative	3 (15)	3 (12)	
Occlusive	1 (5)	2 (8)	
Index stent type			0.44
Bare-metal stent	15 (75)	22 (88)	
Drug-eluting stent	5 (25)	3 (12)	
Index stent diameter, mm	2.97 ± 0.38	3.01 ± 0.47	0.64
Index stent length, mm	33.5 ± 14.5	28.6 ± 13.3	0.26

^a^Classified according to the Mehran classification

Angiographic and procedural success were 100 % in both groups. One patient in the DIOR and 2 patients in the IN.PACT Falcon group received additional stenting during PCI, resulting in device success rates of, respectively, 95 and 92 %. Bailout stent implantation was required to address coronary artery dissection at the proximal edge of the old stent (*n* = 2, including the single DIOR patient) and to treat residual significant stenosis just before the proximal stent edge (*n* = 1). In the DIOR group, there was a trend towards use of larger caliber predilatation balloons (3.1 ± 0.4 vs. 2.9 ± 0.4 mm, *p* = 0.07) and shorter DEB (27.3 ± 9.4 vs. 32.4 ± 12.0 mm, *p* = 0.10), the latter reflecting shorter lesions. Details on procedural data are provided in Table 3.

**Table 3:** Procedural features

	DIOR (*n* = 20)	IN.Pact Falcon (*n* = 25)	*p* value
Predilatation with standard balloon	20 (100)	24 (96)	0.37
Predilatation balloon diameter, mm	3.1 ± 0.4	2.9 ± 0.4	0.07
Predilatation balloon length, mm	17.4 ± 5.4	20.7 ± 7.9	0.26
Predilatation pressure, ATM	14.8 ± 4.5	14.2 ± 5.0	0.71
>1 DEB used per lesion	2 (10)	6 (24)	0.27
DEB diameter, mm	3.2 ± 0.4	3.1 ± 0.3	0.34
2.50	2 (10)	1 (4)	
2.75	0	3 (12)	
3.00	9 (45)	13 (52)	
3.50	8 (40)	8 (32)	
4.00	1 (5)	0	
DEB length, mm	27.3 ± 9.4	32.4 ± 12.0	0.10
DEB inflation pressure, ATM	10.8 ± 3.0	11.3 ± 3.0	0.54
DEB inflation time, s	58.0 ± 5.3	52.0 ± 13.2	0.18
Postdilatation with standard balloon	3 (15)	1 (4)	0.31
Maximum balloon diameter to index stent diameter ratio	1.09 ± 0.08	1.07 ± 0.09	0.34
Additional stenting	1 (5)	2 (8)	0.69
Angiographic success	20 (100)	25 (100)	
Device success	18 (90)	23 (92)	0.69
Procedural success	20 (100)	25 (100)	

### Angiographic outcomes and clinical follow-up

**Fig. 1:**
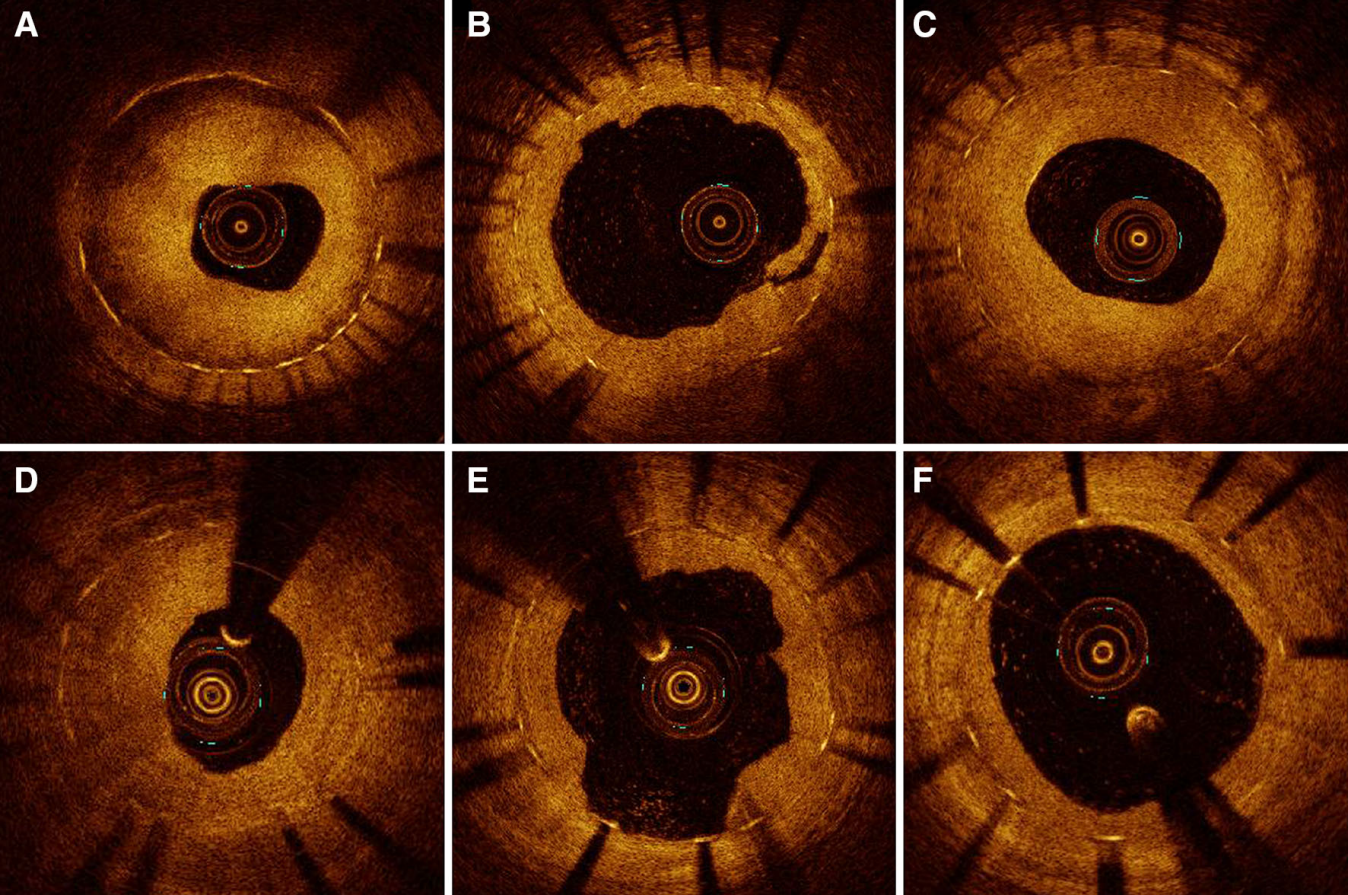
Illustrative OCT imaging of the same coronary segment for each time point (baseline, post-PCI and follow-up). Severe in-stent restenosis at baseline (**a** and **d**). After PCI, lumen enlargement with neointimal disruption and (micro) dissections are observed (**b** and **e**), caused by the mechanical effect of DEB angioplasty. Follow-up shows complete healing of the dissections with a moderate increase (*C*) and limited decrease in neointima (*F*)

Angiographic data are presented in Table 4. Baseline angiographic characteristics were similar between the groups. In-segment LLL was significantly smaller for the IN.PACT Falcon than for the DIOR (−0.03 ± 0.43 vs. 0.36 ± 0.48 mm, *p* = 0.014). The cumulative distribution of in-segment LLL is depicted in Fig. 1 for both DEB. In-segment diameter stenosis at follow-up was lower in IN.PACT Falcon-compared to DIOR-treated patients (30.7 ± 16.2 vs. 41.3 ± 22.6 %, *p* = 0.083), approaching statistical significance. Binary restenosis (both in-stent and in-segment) rate was 39 % in the DIOR and 17 % in the IN.PACT Falcon group (*p* = 0.16).

**Table 4:** Quantitative angiography and fractional flow reserve measurements

	DIOR (*n* = 20)	IN.Pact Falcon (*n* = 25)	*p* value
Preprocedural			
Reference vessel diameter, mm	2.32 ± 0.51	2.35 ± 0.46	0.82
Minimal lumen diameter, mm	0.59 ± 0.28	0.58 ± 0.38	0.98
Diameter stenosis, %	75.0 ± 12.5	75.3 ± 16.1	0.94
Lesion length, mm	23.7 ± 9.5	26.4 ± 12.6	0.52
Fractional flow reserve	18 (90)	22 (88)	
Distal of the stent	0.65 ± 0.11	0.58 ± 0.17	0.31
In-stent gradient	0.33 ± 0.12	0.37 ± 0.18	0.54
Postprocedural			
Minimal lumen diameter, mm	1.88 ± 0.62	1.83 ± 0.47	0.79
Diameter stenosis, %	20.3 ± 9.17	27.5 ± 15.9	0.20
Acute gain, mm	1.29 ± 0.50	1.26 ± 0.61	0.85
Residual binary stenosis	0	2 (8)	0.50
Fractional flow reserve	20 (100)	25 (100)	
Distal of the stent	0.93 ± 0.05	0.92 ± 0.05	0.35
In-stent gradient	0.05 ± 0.03	0.06 ± 0.04	0.40
Follow-up	18 (90)	23 (92)	
Minimal lumen diameter in-stent, mm	1.46 ± 0.68	1.83 ± 0.62	0.064
Minimal lumen diameter in-segment, mm	1.41 ± 0.66	1.69 ± 0.56	0.11
Diameter stenosis in-stent, %	40.1 ± 23.9	26.0 ± 18.3	0.049
Diameter stenosis in-segment, %	41.3 ± 22.6	30.7 ± 16.2	0.083
Late lumen loss in-stent, mm	0.41 ± 0.54	0.01 ± 0.43	0.026
Late lumen loss in-segment, mm	0.36 ± 0.48	-0.03 ± 0.43	0.014
Binary restenosis in-stent	7 (39)	4 (17)	0.16
Binary restenosis in-segment	7 (39)	4 (17)	0.16
Fractional flow reserve	18 (90)	23 (92)	
Distal of the stent	0.84 ± 0.13	0.92 ± 0.07	0.029
In-stent gradient	0.13 ± 0.12	0.05 ± 0.05	0.002
6-months clinical outcome	20 (100)	25 (100)	
Cardiac death	0	0	
Myocardial infarction	0	0	
Stent thrombosis	0	0	
Target lesion revascularization	7 (35)	2 (8)	0.057

Clinical follow-up at 6-months was available for all patients (Table 4). There were no cases of cardiac death, myocardial infarction or stent thrombosis during follow-up. A strong trend towards a higher TLR rate was observed for the DIOR as compared to the IN.PACT Falcon (35 vs. 8 %, *p* = 0.057). Of the 7 DIOR and 2 IN.PACT Falcon patients that received TLR, respectively 2 (29 %) and 1 (50 %) presented with DES-ISR at baseline. All lesions treated with TLR were FFR positive.

### Fractional flow reserve and optical coherence tomography

Fractional flow reserve measurements (Table 4) were performed in 89 % (*n* = 40), 100 % (*n* = 45) and 91 % (*n* = 41) of patients at baseline, postprocedure and follow-up, respectively. Preprocedure FFR was forgone in five patients as the FFR wire could not pass the target lesion, postprocedure FFR was performed in all patients. FFR at follow-up was missing in four patients who denied angiographic follow-up. Baseline and postprocedure FFR distal of the stent were comparable between the groups (*p* = 0.31 and *p* = 0.35, respectively), as were in-stent FFR gradients (*p* = 0.54 and *p* = 0.40, respectively). At follow-up, FFR distal of the stent was significantly higher in the IN.PACT Falcon group (0.92 ± 0.07 vs. 0.84 ± 0.13, *p* = 0.029) and in-stent FFR gradient was lower (0.05 ± 0.05 vs. 0.13 ± 0.12, *p* = 0.002).

**Fig. 2:**
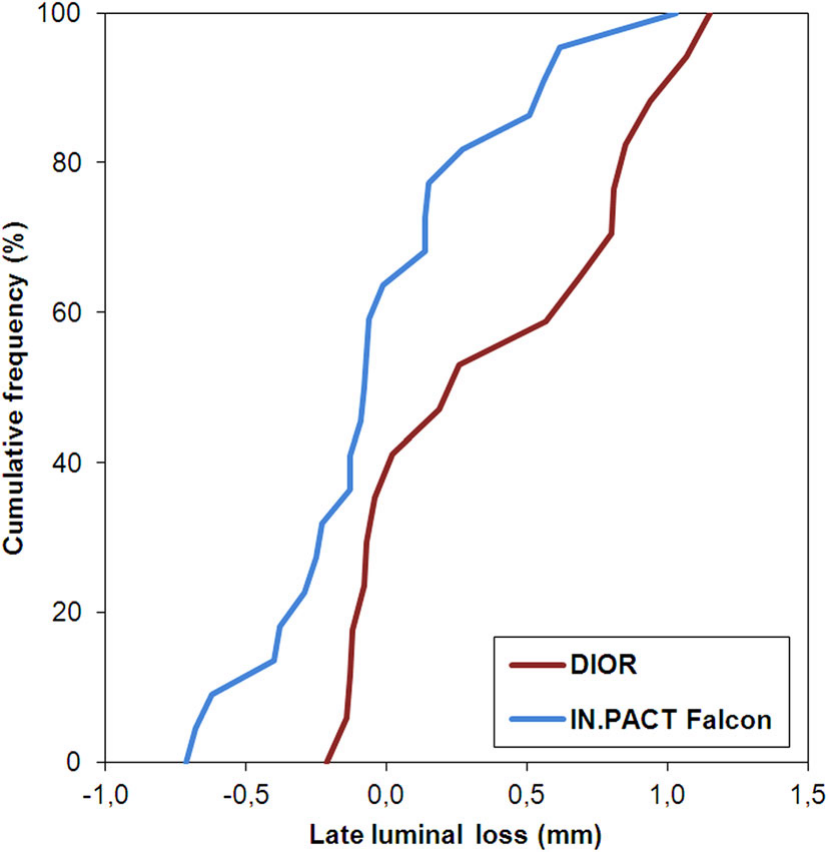
Cumulative distribution of in-segment late lumen loss for the DIOR and IN.PACT Falcon

Overall, preprocedural, postprocedural, and follow-up OCT (Table 5; Fig. 2) were available in 78 % (*n* = 35), 96 % (*n* = 43), and 87 % (*n* = 39) of patients, respectively, with no differences between the groups. Reasons for missing OCT data were: the inability to cross the target lesion (*n* = 8) and poor image quality (*n* = 2), at baseline, technical issues with the acquisition catheter (*n* = 1) and poor image quality (*n* = 1) just after the procedure, and the refusal of follow-up angiography (*n* = 4) and poor image quality (*n* = 2), at follow-up. Baseline OCT derived lumen, neointima and stent characteristics were well matched between the groups. Postprocedure, mean and minimal stent area were significantly smaller in the DIOR group (both *p* = 0.02), while residual neointimal burden was comparable among the groups (similar mean and maximum percentage neointimal area stenosis and percentage of stent volume occupied by neointima). At follow-up, mean and maximum neointimal area stenosis as well as neointima occupied stent volume were larger for the DIOR group (all *p* < 0.05) with concomitant smaller minimal and mean lumen area (both *p* < 0.01). Stent strut analysis is depicted in Supplementary Table 1 (Online Resource 1). In the IN.PACT Falcon group, a small, but significantly larger portion of overall uncovered stent struts was detected at follow-up (*p* = 0.001), as practically all struts in the DIOR group were covered.

**Table 5:** Optical coherence tomographic cross-section analysis

	DIOR (*n* = 20)	IN.pact falcon (*n* = 25)	*p* value
Preprocedure	16 (80)	19 (76)	
Stent length analyzed, mm	23.9 [20.1–30.0]	22.0 [15.4–35.7]	0.29
Mean lumen area, mm^2^	3.1 [2.3–4.5]	3.6 [2.7–4.5]	0.32
Minimal lumen area, mm^2^	1.1 [0.7–1.4]	1.3 [0.9–1.8]	0.13
Mean stent area, mm^2^	6.5 [5.6–8.5]	7.2 [6.2–8.9]	0.31
Minimal stent area, mm^2^	5.3 [3.6–7.2]	5.4 [4.4–7.1]	0.32
Mean neointimal area stenosis, %	50.7 [35.4–66.1]	53.0 [43.7–58.9]	0.90
Maximum neointimal area stenosis, %	80.4 [74.2–88.7]	82.8 [73.6–85.9]	0.79
Lumen volume, mm^3^	89.7 [45.8–103.4]	77.4 [60.5–129.1]	0.92
Stent volume, mm^3^	156 [132–227]	176 [124–217]	0.92
Neointimal volume, mm^3^	66.4 [53.8–134]	87.4 [69.4–102]	0.53
Neointima occupied stent volume, %	51.5 [34.9–65.1]	53.2 [40.8–58.1]	0.95
Postprocedure	20 (100)	25 (100)	
Stent length analyzed, mm	23.9 [19.7–29.9]	21.8 [15.6–32.0]	0.40
Mean lumen area, mm^2^	5.0 [4.1–6.8]	6.2 [5.5–7.1]	0.093
Minimal lumen area, mm^2^	3.3 [2.6–4.7]	4.7 [3.0–5.4]	0.10
Mean stent area, mm^2^	7.4 [6.0–9.7]	9.8 [8.8–11.0]	0.016
Minimal stent area, mm^2^	6.1 [4.1–8.5]	8.2 [7.5–9.2]	0.024
Mean neointimal area stenosis, %	28.3 [24.4–40.4]	33.5 [30.3–36.8]	0.58
Maximum neointimal area stenosis, %	47.8 [37.8–55.2]	48.3 [43.1–50.8]	0.96
Neointima occupied stent volume, %	28.2 [24.8–40.2]	33.6 [29.0–37.4]	0.38
Follow-up	17 (85)	22 (88)	
Stent length analyzed, mm	23.8 [21.2–31.7]	22.4 [18.1–32.8]	0.28
Mean lumen area, mm^2^	4.6 [3.4–6.1]	6.0 [5.2–7.8]	0.008
Minimal lumen area, mm^2^	2.4 [1.8–3.7]	4.0 [3.1–6.0]	0.009
Mean stent area, mm^2^	7.6 [6.1–9.5]	9.1 [7.5–11.5]	0.066
Minimal stent area, mm^2^	6.5 [4.4–8.0]	7.7 [5.3–9.8]	0.16
Mean neointimal area stenosis, %	42.8 [23.7–55.3]	31.6 [24.9–37.5]	0.011
Maximum neointimal area stenosis, %	66.4 [49.9–76.6]	47.7 [37.3–60.7]	0.010
Neointima occupied stent volume, %	41.7 [26.0–55.1]	30.5 [23.7–36.5]	0.047

**Fig. 3:**
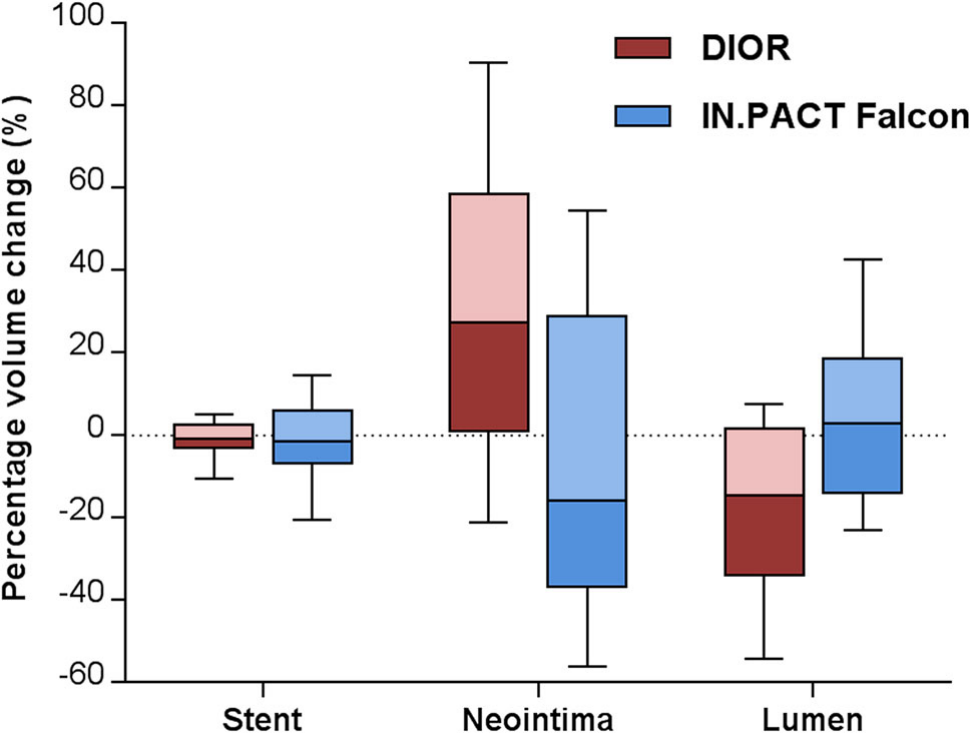
Percentage changes in postprocedure and follow-up in stent, neointima and lumen volumes derived from optical coherence tomography (*negative values* represent a decrease and *positive values* an increase in volumes during follow-up)

Table 6 shows the percentage changes in postprocedure and follow-up measurements. A percentage decrease in minimal lumen diameter was observed for the DIOR, while the IN.PACT Falcon showed a relative increase (*p* = 0.034). Percentage volume changes revealed an overall decrease in lumen and increase in neointima in the DIOR group, as opposed to an increase in lumen and decrease in neointima in the IN.PACT Falcon group (Fig. 3). A significant difference was observed among the DEB in the percentage change of in-stent FFR gradient between postprocedure and follow-up, demonstrating an in-stent gradient increase for the DIOR and a decrease for the IN.PACT Falcon (*p* = 0.003). The difference in neointimal growth and lumen volume change on OCT remained statically significant after adjusting for hypercholesterolemia, postdilatation, and postprocedure minimal lumen area on OCT. Comparable differences were observed when focusing only on BMS-ISR (see Supplementary Table 2, Online Resource 1).

**Table 6:** Percentage changes between postprocedure and follow-up

	DIOR (*n* = 20)	IN.pact falcon (*n* = 25)	Crude *p* value	Adjusted *p* value*
Quantitative coronary angiography	18 (90)	23 (92)		
Minimal lumen diameter change, %	–10.4 [–42.3 to 4.7]	4.8 [−7.7 to 16.8]	0.034	0.26
Diameter stenosis change, %	28.6 [−7.1 to 187]	2.6 [−49.7 to 67.6]	0.032	0.32
Optical coherence tomography	17 (85)	21 (84)		
Minimal lumen area change, %	−30.2 [−49.5 to 2.6]	−13.4 [−21.8 to 19.4]	0.048	0.097
Maximal neointimal area change, %	33.8 [6.2 to 72.0]	−8.9 [−21.0 to 33.0]	0.002	0.007
Maximal neointimal area stenosis change, %	35.8 [8.7 to 59.1]	14.6 [−21.7 to 36.6]	0.014	0.009
Lumen volume change, %	−14.6 [−34.0 to 1.7]	2.89 [−14.0 to 18.6]	0.011	0.026
Stent volume change, %	−0.7 [−3.0 to 2.7]	−1.6 [−6.9 to 5.9]	0.67	0.77
Neointimal volume change, %	27.2 [1.1 to 58.6]	−15.8 [−36.7 to 28.3]	0.006	0.028
Fractional flow reserve	17 (85)	22 (88)		
FFR stent gradient change, %	69.0 [0.0 to 238]	−40.8 [−58.9 to 18.8]	0.003	0.46

* Adjusted for differences in hypercholesterolemia, postdilatation and postprocedure OCT minimal lumen area

## Discussion

The aim of this study was to compare the antirestenotic efficacy (both morphological and functional) of two established DEB with different coatings in the treatment of ISR: the urea-paclitaxel coated IN.PACT Falcon and the shellac-paclitaxel coated DIOR. Comparative assessment of antirestenotic efficacy was performed by means of a unique investigational approach, comprising serial morphological (QCA and OCT) and functional (FFR) evaluation of the target lesion.

The most important findings of this study are: (1) the magnitude of neointimal inhibition is larger for the IN.PACT Falcon than the DIOR DEB in the treatment of ISR as demonstrated by the lower angiographic LLL and decrease in neointimal volume on OCT at follow-up; (2) the morphological changes detected in the IN.PACT Falcon group show favorable hemodynamics according to FFR measurements; (3) the neointimal suppression observed for the IN.PACT Falcon leads to less re-ISR compared to the DIOR as shown by the trend towards lower diameter stenosis and TLR rate.

### Comparison with previous data

Antirestenotic efficacy was higher for the IN.PACT Falcon compared to the DIOR, which resulted in a benefit in clinical outcome. The present results for the IN.PACT Falcon are in line with those previously reported for this particular DEB. A small prospective study on BMS-ISR observed an in-segment LLL of 0.02 ± 050 mm and TLR rate of 4.3 % at 6 months [20]. In this regard, the IN.PACT Falcon compares favorably to other benchmark DEB in the setting of ISR [10, 21]. The findings for the DIOR are on odds with prior data. In the Valentines-I trial, a prospective study on the efficacy of the DIOR in both DES-ISR and BMS-ISR, an encouraging 7.4 % TLR rate was found after a mean follow-up of 7.5 months [22]. The lack of mandatory angiographic and FFR follow-up may explain the discrepancy with the present study findings.

Notably, the larger proportion of drug-eluting index stents in the DIOR group had raised some concern for bias, as recurrent restenosis is more common in DES-ISR compared to BMS-ISR [2, 22]. Therefore, a subanalysis in BMS-ISR patients was performed, which showed results identical to the overall population (supplementary Table 2, Online Resource 1). Other factors that have been associated with recurrent restenosis, i.e., lesion length and ISR pattern [23], were well balanced between the groups. Study design did not account for unmeasured confounding factors, such as the pathophysiology and tissue characteristics of ISR. Both features are closely related to the type of index stent, however [24].

### Importance of DEB coating technology

The present study confirms, for the first time using mandatory angiographic follow-up, that no class effect can be assumed for DEB in the clinical setting. An earlier report from the SCAAR (Swedish Coronary and Angioplasty) registry has shown important differences in restenosis between two commonly used DEB [2]. Of note, the DEB compared in SCAAR were very dissimilar with respect to the employed modifications to improve drug release, as the DEB demonstrating the worse outcome forgoes the use of an excipient. The pivotal role of excipients in DEB was demonstrated before in preclinical studies [13, 14] and is reflected in the ambiguous results of clinical trials in small vessel disease [16, 17].

Despite both DEB in our study are equipped with an excipient-based coating formulation, large differences in antirestenotic efficacy are observed. This is best expressed by the percentage changes in neointimal volume during follow-up, revealing a 30 % increase for the DIOR and a 16 % decrease for the IN.PACT Falcon (Fig. 2). Where the IN.PACT Falcon not only inhibits neointimal formation, but also induces positive remodeling, the DIOR fails to suppress neointimal growth to a sufficient degree. Positive remodeling after DEB treatment may result from neointimal smooth muscle cell loss due to paclitaxel-induced apoptosis or necrosis, two phenomena that require high local tissue concentrations of drug [25]. Otherwise, cicatricial shrinkage of neointima through healing of the barotrauma-induced dissections may be involved [19]. Regardless of the underlying mechanism, the net effect of these processes only becomes apparent as positive remodeling in the presence of sufficient neointimal suppression.

The observed disparity in antirestenotic efficacy may be attributed to the difference in excipients, since all other DEB characteristics were constant for both devices. Animal studies have suggested before that the use of an excipient in a DEB coating is no guarantee for effective drug transfer to the vessel wall [14]. However, early paclitaxel tissue concentrations measured during the preclinical testing of the DIOR were similar to those reported for other established DEB [26, 27].

According to more recent data, it may not be the acute transfer of drug, but the durability of paclitaxel activity that falls short in the DIOR. Using histological vascular healing parameters (inflammation-, fibrin- and smooth muscle cell loss score) as a proxy, the drug effect in DIOR treated porcine arteries was observed to disappear rapidly as it was already absent at one month follow-up [28]. This is unsettlingly fast, as the vascular healing signs associated with DEB well-known for their effectiveness remain even detectable up to several months [29]. Thus, although effective drug uptake does occur in DIOR angioplasty, its effect appears short-lived. The cause of this unfavorable pharmacokinetic profile of the DIOR remains speculative, but may reside in an undesired drug-excipient interaction that may lead to: (1) a decreased binding of paclitaxel to non-specific binding sites accelerating its tissue clearance, (2) a chemical configuration of paclitaxel that interferes with its metabolic activity, or (3) less homogeneous release of paclitaxel resulting in unfavorably large spatial differences in tissue drug concentration. Interestingly, the above data supports the longstanding presence of active drug as the mechanism responsible for durable neointimal inhibition by DEB, rather than the prevention of early growth initiating events [27].

### Investigational model

This study is the first in-human comparative assessment of DEB using highly sensitive techniques to quantify morphological and functional changes at the target lesion site. The unique investigational approach ensued, allowed for a precise and reliable assessment of antirestenotic efficacy.

While angiography is commonly used to assess neointimal inhibition after DEB treatment, it remains lumenography: an imaging technique providing data on lumen diameter change at follow-up without differentiating between the underlying mechanisms (neointimal growth or stent recoil). In contrast, morphological assessment by OCT provides separate measurements on lumen, neointimal and stent dimensions. This enables the precise calculation of neointimal dimension changes between specified time points, irrespective of changes in stent volume. Moreover, the OCT derived neointimal volume change at follow-up represents neointimal inhibition along the entire lesion length, whereas its angiographic counterpart (LLL) may only reflect local neointimal suppression. Neointimal volume change on OCT may thus provide a more honest assessment of antirestenotic potency, although focal lumen changes may be ultimately responsible for recurrent restenosis.

Finally, structural functional assessment at follow-up by means of FFR allowed for objective evaluation of the impact of morphological changes on vessel patency. This approach is highly recommendable, since a poor correlation exists between angiographic assessment of moderate or diffuse type ISR and the hemodynamic significance of lesions assessed by FFR [30]. FFR confirmed the better antirestenotic efficacy for the IN.PACT Falcon observed on morphological data.

## Limitations

Although baseline data were well matched between the groups, the non-randomized design is an important limitation of this study. Statistical correction for differences in baseline characteristics was omitted because of the small patient number in each treatment group. Notwithstanding the unequivocal results of the exhaustive analysis of antirestenotic efficacy in this study, our findings should be interpreted in light of the small sample size. Although no blinding was provided for QCA and OCT analysis, there was only limited opportunity for observer bias as the analyses were performed semi-automatically.

## Conclusions

In this study comparing two commonly used DEB in the treatment of ISR, the IN.PACT Falcon demonstrated higher antirestenotic efficacy than the DIOR on both morphological and functional parameters. This differential efficacy is probably caused by the different excipients in the DEB coatings, leading to different pharmacokinetic profiles. Notably, both of the investigated devices have previously shown antirestenotic benefit in preclinical and clinical studies. Therefore, direct comparative assessment of DEB in the clinical setting seems warranted, as currently available DEB may represent a spectrum of devices with an antirestenotic efficacy anywhere between a standard uncoated balloon and a ‘true’ DEB.

## Electronic supplementary material

Below is the link to the electronic supplementary material.

Supplementary material 1 (PDF 221 kb)
