# Supplementary material for: Comparative assessment of the antirestenotic efficacy of two paclitaxel drug-eluting balloons with different coatings in the treatment of in-stent restenosis
Source: Clin Res Cardiol. 2015 Nov 5;105:401–11. doi: 10.1007/s00392-015-0934-0 (PMC4841852; doi:10.1007/s00392-015-0934-0)
Supplement: Supplementary file 1 — Supplementary material 1 (PDF 221 kb) [file 392_2015_934_MOESM1_ESM.pdf]

ELECTRONIC SUPPLEMENTARY MATERIAL

Comparative Assessment of the Antirestenotic Efficacy of Two Paclitaxel Drug-Eluting Balloons with Different Coatings in the Treatment of In-Stent Restenosis

Clinical Research in Cardiology

Freek Nijhoff, MD*; Pieter R. Stella, MD, PhD*; Maartje S. Troost, MD*; Anouar Belkacemi, MD, PhD†; Hendrik M. Nathoe, MD, PhD*; Michiel Voskuil, MD, PhD*; Mariam Samim, MD, PhD* ; Pieter A. Doevendans, MD, PhD*; Pierfrancesco Agostoni, MD, PhD*

* Department of Cardiology, University Medical Center Utrecht, Utrecht, Netherlands

† Department of Cardiology, Isala Clinics, Zwolle, The Netherlands

Contents

Supplementary Methods, p. 2

Supplementary Tables, p. 4

Supplementary References, p. 6

Address of correspondence:

Pierfrancesco Agostoni, MD, PhD

Department of Cardiology, University Medical Center Utrecht

Heidelberglaan 100, room E.04.210, 3584 CX, Utrecht, The Netherlands

Phone: +31 88 755 6167; Fax: +31887555427; Email: agostonipf@gmail.com

SUPPLEMENTARY METHODS

Devices

The paclitaxel DEB used in this study are the DIOR II (EuroCor GmbH, Bonn, Germany) and IN.PACT Falcon (Medtronic Vascular Inc., Santa Rosa, CA, USA), both devices have been described in detail elsewhere [1, 2]. Both DEB consist of a semi-compliant angioplasty balloon which is loaded with paclitaxel in a concentration of 3 µg/mm^2 balloon surface. The DIOR balloon coating contains shellac (a natural resin composed of shellolic and alleuritic acid) as excipient in a 1:1 mixture with paclitaxel and is applied to the balloon surface through a micro-pipetting procedure. The IN.PACT Falcon is coated with the FreePac™ hydrophilic coating formulation, which contains the naturally occurring urea as excipient, by a dispersion method. Recommended inflation time is 30 to 60 seconds for both DEB to allow sufficient drug transfer to the vessel wall.

Quantitative coronary angiography, optical coherence tomography and fractional flow reserve

All study investigations were performed before the procedure, immediately after the procedure and at 6-months follow-up. Quantitative Coronary Angiography (QCA) and Optical Coherence Tomography (OCT) images were analyzed by an independent operator. Dedicated software (CAAS 5.9.1 Research Edition, Pie Medical Imaging, Maastricht, The Netherlands) was used to perform QCA in a standardized fashion [3]. In the preprocedure, postprocedure and follow-up images, the stent(s) and additional 5 mm proximal and distal to the stent(s) edges were analyzed. Lesion length and minimal lumen diameter were directly determined by the QCA software, whereas reference vessel diameter was estimated by an interpolation method. Percent diameter stenosis was subsequently computed. Binary restenosis was defined as diameter stenosis ≥50% at angiographic follow-up. Late lumen loss was defined as the difference in postprocedure and follow-up minimal lumen diameter in the same segment (proximal to the stent, in-stent, distal to the stent or in-segment).

OCT was performed using time-domain (M3, LightLab Imaging, MA, USA) or frequency domain systems (C7XR, St. Jude Medical, MA, USA) after intracoronary administration of 200 µg of nitroglycerin. Images were acquired under continuous infusion of contrast from the guiding catheter by means of a controlled injection (2-4 ml/s with 200-300 psi, depending on the coronary assessed), as advocated [4]. Automated pullback speed and frame rate were 2 mm/s and 20 fps for the M3 and 20 mm/s and 100 fps for the C7XR system, respectively. All cross-sectional images (frames) were screened for quality and excluded if reliable analysis was impeded by residual blood, side-branches, imaging artifacts or reverberation, or if any portion of the stent was out of the screen. Dedicated software (Curad BV, Amsterdam, The Netherlands) including automated contour detection algorithms

was used for image analysis. Two contours were automatically delineated: the lumen contour (for each frame) and the stent contour (every 2 frames for the C7XR and every 5 frames for the M3 system, corresponding with every 0.4 and 0.5 mm, respectively). In-between frames an automated contour interpolation was performed. Manual corrections were applied where needed. Lumen, stent and neointima dimensions were calculated by the software. Stent struts were semi-automatically detected and classified as: covered embedded, covered protruding, uncovered apposed, and malapposed. Definitions and strut classification were outlined in a previous publication [1].

Fractional flow reserve was measured with a coronary pressure wire (Certus Pressure Wire, St. Jude Medical, St. Paul, Minnesota) under maximal hyperemia induced by adenosine infusion in a central vein (at a rate of 140 μg/Kg/min-1). After positioning of the FFR wire as distal as possible in the target vessel, FFR was assessed at 3 positions by means of a manual pullback: at the distal part of the vessel, just at the level of the distal stent edge and just at the level of the proximal stent edge. An in-stent gradient was calculated as the difference between FFR at the distal and proximal stent edges. Quality of the FFR measurements was checked by obtaining the FFR at the level of the guiding catheter (which should be 1.00).

Follow-up and endpoints

Angiographic follow-up was scheduled per protocol at 6 months, unless indicated earlier on clinical grounds. Clinical follow-up was obtained simultaneously with angiography or by telephone interviews at 6 months. All clinical events were documented after careful examination of relevant hospital files.

Antirestenotic efficacy, the outcome of interest, refers to the potency of the DEB to inhibit neointimal growth, not to the prevention of clinical restenosis per se. Main endpoints of this study were angiographic in-segment late luminal loss (LLL) and diameter stenosis, percentage changes in FFR and OCT parameters, and clinical outcomes according to the Academic Research Consortium criteria. Target lesion revascularization (TLR) was defined as any repeat intervention (percutaneous or surgical) to address recurrent restenosis of the treated segment (including the stent and the proximal and distal 5 mm segments). Revascularization of a target lesion was clinically indicated in case of restenosis >50% associated with angina pectoris and/or objective signs of ischemia (stress test or FFR), or in case of restenosis >70% in the absence of the above mentioned signs or symptoms. Angiographic success was defined as attainment of final residual stenosis <30% (by visual estimation) and TIMI 3 flow, using any percutaneous technique. Device success was defined as achievement of angiographic success by using the DEB device (no additional stenting allowed). Procedural success was defined as angiographic success in the absence of any in-hospital major adverse cardiac events.

SUPPLEMENTARY TABLES

Table 1. Stent strut analysis on optical coherence tomography.

	**DIOR II (n=20)**	**In.Pact Falcon (n=25)**	**p-value**
Preprocedure	16 (80)	19 (76)	
Total number of stent struts analyzed	9510	8094	
Covered embedded struts per lesion, %	98.9 [93.3-100]	100 [97.6-100]	0.22
Covered protruding struts per lesion, %	0.9 [0.0-6.1]	0.0 [0.0-1.1]	0.11
Uncovered struts per lesion, %	0.0 [0.0-0.3]	0.0 [0.0-0.5]	0.65
Malapposed struts per lesion, %	0.0 [0.0-0.2]	0.0 [0.0-0.4]	0.45
Covered struts overall, %	100 [99.1-100]	100 [99.3-100]	0.94
Uncovered struts overall, %	0.0 [0.0-0.9]	0.0 [0.0-0.7]	0.93
Postprocedure	20 (100)	25 (100)	
Total number of stent struts analyzed	11827	7676	
Covered embedded struts per lesion, %	96.1 [91.7-99.0]	97.2 [92.4-98.9]	0.90
Covered protruding struts per lesion, %	2.2 [0.4-6.2]	0.5 [0.0-1.0]	0.002
Uncovered struts per lesion, %	0.7 [0.0-1.2]	1.9 [0.6-6.7]	0.011
Malapposed struts per lesion, %	0.1 [0.0-0.6]	0.0 [0.0-0.2]	0.49
Covered struts overall, %	99.0 [98.1-100]	98.1 [92.4-98.9]	0.022
Uncovered struts overall, %	1.0 [0.0-2.0]	1.9 [1.1-7.6]	0.022
Follow-up	17 (85)	22 (88)	
Total number of stent struts analyzed	10660	8525	
Covered embedded struts per lesion, %	100 [92.9-100]	97.7 [93.6-99.4]	0.072
Covered protruding struts per lesion, %	0.0 [0.0-6.8]	0.7 [0.0-1.7]	0.41
Uncovered struts per lesion, %	0.0 [0.0-0.2]	0.9 [0.0-3.1]	0.003
Malapposed struts per lesion, %	0.0 [0.0-0.0]	0.2 [0.0-0.2]	0.016
Covered struts overall, %	100 [99.7-100]	98.7 [96.5-99.8]	0.001
Uncovered struts overall, %	0.00 [0.0-0.3]	1.3 [0.2-3.5]	0.001

Table 2. Percentage changes between postprocedure and follow-up for bare-metal ISR only.

	**DIOR II (n=15)**	**In.Pact Falcon (n=22)**	**p-value**
Quantitative coronary angiography	13 (87)	20 (91)	
Minimal lumen diameter change, %	-17.3 [-41.1-5.0]	8.2 [-5.3-17.9]	0.022
Diameter stenosis change, %	40.8 [-26.4-111]	-15.0 [-51.9-22.6]	0.083
Optical coherence tomography	12 (80)	18 (82)	
Minimal lumen area change, %	-30.2 [-49.5-2.6]	-13.4 [-21.8-19.4]	0.022
Maximal neointimal area change, %	30.7 [4.9-59.7]	-9.5 [-19.4-30.2]	0.014
Maximal neointimal area stenosis change, %	33.3[-0.9-55.0]	6.4 [-22.8-32.2]	0.034
Lumen volume change, %	-17.3 [-37.7--3.0]	6.1 [-11.7-24.8]	0.001
Stent volume change, %	-1.5 [-3.6-1.4]	0.2 [-5.9-6.7]	0.37
Neointimal volume change, %	29.2 [2.4-45.7]	-15.8 [-36.7-28.3]	0.005
Fractional flow reserve	13 (87)	19 (86)	
FFR stent gradient change, %	48.2 [0.0-124]	-41.7 [-57.8-0.0]	0.003

SUPPLEMENTARY REFERENCES

[1] Agostoni P, Belkacemi A, Voskuil M, Nathoe HM, Doevendans PA, Stella PR. Serial morphological and functional assessment of drug-eluting balloon for in-stent restenotic lesions: mechanisms of action evaluated with angiography, optical coherence tomography, and fractional flow reserve. J Am Coll Cardiol Interv. 2013;6:569-76.

[2] Posa A, Nyolczas N, Hemetsberger R, et al. Optimization of drug-eluting balloon use for safety and efficacy: evaluation of the 2nd generation paclitaxel-eluting DIOR-balloon in porcine coronary arteries. Catheter Cardiovasc Interv. 2010;76:395-403.

[3] Garrone P, Biondi-Zoccai G, Salvetti I, et al. Quantitative coronary angiography in the current era: principles and applications. J Interv Cardiol. 2009;22:527-36.

[4] Prati F, Cera M, Ramazzotti V, et al. From bench to bedside: a novel technique of acquiring OCT images. Circ J 2008;72:839-43.
